# Malaria control in Timor-Leste during a period of political instability: what lessons can be learned?

**DOI:** 10.1186/1752-1505-3-11

**Published:** 2009-12-16

**Authors:** Joao S Martins, Anthony B Zwi, Nelson Martins, Paul M Kelly

**Affiliations:** 1 School of Public Health and Community Medicine, The University of New South Wales, Sydney, Australia; 2 Ministry of Health, Dili, Timor-Leste; 3 National Centre for Epidemiology & Population Health, College of Medicine, Biology & Environment, Australian National University, Canberra, Australia; 4 Universidade da Paz, Manleuana, Dili, Timor-Leste

## Abstract

**Background:**

Malaria is a major global health problem, often exacerbated by political instability, conflict, and forced migration.

**Objectives:**

To examine the impact of political upheaval and population displacement in Timor-Leste (2006) on malaria in the country.

**Method:**

Case study approach drawing on both qualitative and quantitative methods including document reviews, in-depth interviews, focus group discussions, site visits and analysis of routinely collected data.

**Findings:**

The conflict had its most profound impact on Dili, the capital city, in which tens of thousands of people were displaced from their homes. The conflict interrupted routine malaria service programs and training, but did not lead to an increase in malaria incidence. Interventions covering treatment, insecticide treated nets (ITN) distribution, vector control, surveillance and health promotion were promptly organized for internally displaced people (IDPs) and routine health services were maintained. Vector control interventions were focused on IDP camps in the city rather than on the whole community. The crisis contributed to policy change with the introduction of Rapid Diagnostic Tests and artemether-lumefantrine for treatment.

**Conclusions:**

Although the political crisis affected malaria programs there were no outbreaks of malaria. Emergency responses were quickly organized and beneficial long term changes in treatment and diagnosis were facilitated.

## Background

Globally, malaria poses a threat to approximately 3.3 billion of the world's population with around 250 million clinical cases annually and more than 1 million deaths, mostly in children under 5 years of age [1].

In April and May 2006 serious political instability and violence affected the newly independent Democratic Republic of Timor-Leste. The risk of infectious diseases in conflict-affected settings is increased. Violent conflict causes population displacement and destruction of infrastructure, as well as the reduction or disruption of health services, including routine disease control programs, which can lead to outbreaks [2-5]. Additionally, the lack of clean water supplies, poor sanitation and waste management, overcrowding and poor shelter can increase the risk of communicable diseases including malaria [2,6,7]. The increase of malaria morbidity and mortality due to conflicts have been observed in many conflict areas such as the Democratic Republic of Congo [8], and Afghanistan [9,10]. The increase in malaria incidence in refugees and displaced populations in African countries has been well documented [11].

Malaria has always been one of the biggest public health problems in Timor-Leste. Both *Plasmodium falciparum *and *Plasmodium vivax *are present in the country, although their precise distribution is unknown. Malaria incidence typically increases in the rainy season (November to April). The national cumulative Annual Clinical Malaria Incidence (ACMI) based on syndromic diagnosis in 2005 was 144/1000 population, but varied substantially between districts from 100 to 250 per 1000 population. The Annual Parasite Incidence (API) based on laboratory-confirmed diagnosis in 2006 was 38.5 per 1000 [12]. To support the intervention, the MoH also developed national strategies on malaria control [13] in line with the World Health Organization's Roll Back Malaria Strategy and broader control strategies for mosquito-borne diseases [14]. The Global Fund to fight AIDS, Tuberculosis and Malaria has substantially funded malaria control in Timor-Leste since 2003 [15].

The 2006 crisis originated from alleged ethnically-based discrimination within the military. The aggrieved soldiers, mostly from the west of the country, left their barracks, staged a protest and were dismissed. The detail of the chronology of the 2006 political crisis is outlined elsewhere [16]. Subsequently gang fights and street violence ensued, with over 3000 homes burned down mostly in Dili and displacement of approximately 15% of the country's population. The internally displaced people (IDPs) sought refuge in camps, churches, convents and schools, with some displaced from the capital city, Dili, to districts. In Dili, more than 60 camps were established to provide temporary shelter for displaced people [17].

This study was designed to assess and describe the impact of the 2006 crisis on malaria and critically examine the response by key agencies. It sought to identify key lessons both for Timor-Leste and other similar settings, notably urban areas affected by political instability and displacement.

## Methodology

This case study used both qualitative and quantitative methods. The qualitative methods included document reviews, key informant interviews, focus group discussions and observations. The quantitative data were derived from malaria morbidity data reported from the IDP camps and health facilities to the Ministry of Health (MoH).

Data collection was from September - November 2006 at the same time as for the broader Health Sector Resilience Study [17]. The study was conducted in Dili and four other districts: Aileu, Baucau, Ermera and Lautem. The latter were selected to represent districts affected by the crisis, two each in the East and West of the country. Institutions and individuals selected for this study were identified in consultation with the MoH and were chosen to reflect the range of ways in which districts in different parts of the country might be affected.

Major topics explored in this study included how malaria interventions were organised, the types of malaria interventions delivered during the crisis, the surveillance system used to monitor malaria cases within the IDP camps, the major stakeholders involved in malaria control during the crisis, the implications of the crisis for the malaria control program, and the lessons learned.

Table 1 presents a summary of the methods used and the numbers of in-depth interviews and focus group discussions (FGDs) undertaken. In-depth interviews were held with policy makers and program implementers of the MoH, non-governmental organizations, and United Nations agencies notably the World Health Organization (WHO). Thirty key informants selected on a purposeful basis [18] were interviewed, each interview lasting between thirty minutes and two hours. Interviews were recorded digitally after obtaining consent, and then transcribed in full.

**Table 1:** Summary of qualitative methods used

In-depth interview		Focus group discussion	
***Agency***	***No. people interviewed***	***Participants***	***No. FGDs***

Ministry of Health	8	Camp managers and Site Liaison Officers	1

World Health Organization	3	Health workers	1

Non-governmental organisations	4	Internally displaced persons	1

Cuban Medical Brigade	3		

District Health Services	5		

Government health workers delivering interventions at IDP camps	**7**		

**Total**	**30**		**3**

Three FGDs were held, one with IDP camp managers and Site Liaison Support staff, responsible for addressing the needs of camp populations, the second with health workers, and the third with a group of IDPs. These participants were selected on the basis of either being affected by the crisis and/or being involved in organizing emergency responses. Participants were informed by the researchers at least one week prior to the meeting schedule. The participants of FGDs with health workers and IDPs numbered 12 people, while approximately 40 people attended the 'FGD' with Camp Managers and SLS staff. The latter was more akin to a group meeting, because the researchers were given one hour in the middle of a weekly meeting held at the Ministry of Solidarity and Community Reinsertion, in which to explore issues with those present. Informal observations at three IDP camps and informal discussions with a number of IDPs was also undertaken.

Participants of both in-depth interviews and FGDs were provided with information sheets about the study and informed consent was requested in English or Tetum, the most widely spoken and official language. Interviews were conducted after obtaining signed consent, or verbal consent for those who could not read. No one refused to be interviewed.

Quantitative data on malaria were obtained from the Malaria Unit of the MoH and included aggregate cases diagnosed on a syndromic basis and those cases which had been confirmed with microscopy. Data on ITN distribution were obtained from the MoH and NGOs involved in the net distribution program particularly HealthNet International (HNI), Catholic Relief Service (CRS) and Timor-Leste Servisu Saude Intergradu (TAIS).

### Data analysis

All in-depth interviews and FGDs were transcribed and coded using Nvivo 7 software. Minutes of meetings and relevant documents were reviewed and triangulated with interview and FGD data.

Quantitative data were entered into MS Excel and graphs generated. Malaria incidence rates for Dili district and the rest of the country per 1000 population were calculated for 2004-2007 using the denominator of the 2004 population census figure. Population was based on the 2004 Census; the total country population was 924,624. Population for Dili district was 167, 777. During the crisis an estimated 70,000 people fled out from Dili to Districts in 2006 and 2007. A 'best estimate' of approximately 70,000 was deducted from Dili's population in view of displacement of Dili's residents to districts inside Timor-Leste's territory. The exact number of displaced population from Dili to districts was unknown, estimates have been made ranging 68,000 [19] to 75,000 [17]. The 70,000 used as denominator for this study was drawn from these estimates.

### Ethical clearance

Ethical clearance was obtained from the Human Research Ethics Committee, University of New South Wales (Ref: HREC 06226). In the absence of a formal ethics review structure in Timor-Leste, approval to conduct the study was obtained from the MoH.

## Results

### Malaria morbidity trends

**Figure 1:**
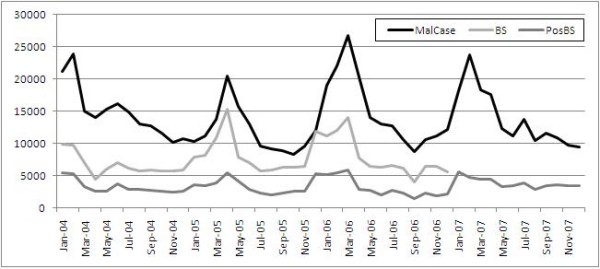
**Monthly national trends of malaria cases in Timor-Leste 2004-2007**. **MalCase **= malaria cases deriving from a combination of syndromic and microscopically confirmed diagnosis; **BS **= Blood Smear carried out to do confirmatory test with light microscopes; **PosBS **= Blood smear positive malaria parasite resulted from the blood test with light microscopes.

Figure 1 describes trends in monthly diagnoses of malaria cases over the period 2004-2007. At country level, there is no indication that the pattern of malaria for 2006 differed substantially from previous years; the peak in early 2006 preceded the instability.

The malaria rates based in Dili District and the rest of the country were estimated from clinically suspected cases reported to the MoH. In Dili and other parts of the country, the malaria rates from May - November are lower than the December - April period. In 2005 and 2006, the rates in Dili were lower than those in the rest of the country. However, in 2007, the rates in Dili, during the May - November period, were higher than those from other parts of the country. This could reflect better surveillance and recording, and/or some decline in control efforts (Table 2). Rates in the rural districts also showed some increases over the previous year during this period, although they were lower than those in Dili.

**Table 2:** Estimates from clinically diagnosed malaria cases (rate per 1000 population) in Dili District and the Rest of the country from 2004-2007.

Month/Year	2004		2005		2006		2007	
	**Dili**	**Rest of the country**	**Dili**	**Rest of the country**	**Dili**	**Rest of the country**	**Dili**	**Rest of the country**

January	28.6	21.7	13.6	10.6	27.5	19.1	30.5	18.4

February	26.2	28.8	22.5	9.9	21.9	24.2	22.8	26

March	19.6	15.6	21.3	13.5	18	31.3	27.8	19

April	24.2	13.1	30	21.3	6.8	25.3	24.5	18.4

May	22.4	15.4	9.3	18.8	2.6	16.7	18.8	12.6

June	21.6	16.6	11	14.9	4.2	15.3	14.5	11.9

July	15.5	16.3	6.4	11.3	4.3	14.9	17.1	14.7

August	10.8	14.8	8.3	10.2	5	12.2	10	11.4

September	14.1	13.7	7.7	10.1	6.3	9.8	18	11.9

October	9.5	13.2	6.3	9.6	6.8	12	17.2	11.1

November	8.2	11.7	5.6	11.5	7.4	12.7	14.5	10.1

December	5.5	13	11.2	13.4	23	12	17.4	9.4

Population was based on 2004 Census; the total country population was 924,624. Population for Dili district was 167, 777, during the crisis an estimated 70,000 people fled out from Dili to Districts in 2006 and 2007.

### Surveillance

**Figure 2:**
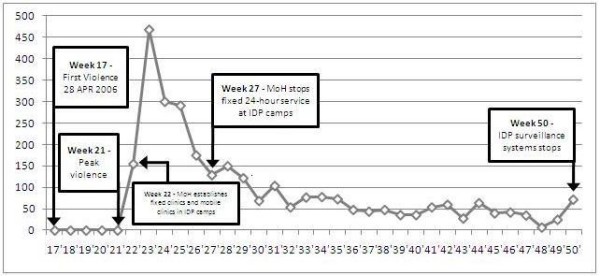
**Weekly trends of suspected malaria from IDP camps in and around Dili, Timor-Leste, from Epidemiological week 22 - 50 in 2006**.

The Surveillance Unit, MoH, continued to monitor 11 diseases with outbreak potential in all health facilities and IDP camps. Although surveillance was in disarray in the early stage of the crisis, the actors involved in the emergency response (MoH surveillance officer, WHO adviser, Cuban Medical Brigade and NGOs) met within weeks to agree on a number of essential diseases that had to be reported to the Surveillance Unit, MoH [17]. As a result, integrated weekly epidemiological surveillance data on these diseases were reported from the last week of May until the third week of December 2006. Surveillance data on suspected malaria cases is presented in Figure 2, showing an increase in June and gradual decrease thereafter. The Figure also indicates the timeline of political instability in 2006 in the country.

In addition, the surveillance activity during the first and second week of the crisis [2^nd ^to 17^th ^June 2006] recorded 17 types of diseases reported: URTI (66%), skin diseases (11%) and both suspected malaria and acute diarrhoea contributing a further 7% each [20].

### Diagnosis and Treatment

Malaria diagnosis in IDP camps relied on a syndromic approach. Laboratory confirmation with microscopy was carried out but was limited to Community Health Centers and hospitals, some of which closed down, temporarily, during the crisis [17].

Malaria treatment followed the standard MoH protocol adopted in 2004. There had been an intensive effort between the MoH and WHO before the crisis to introduce artemisinin-based combination therapy (ACT) for treating falciparum malaria. The WHO ordered around 39,000 doses of artemether-lumefantrine in anticipation of possible outbreaks and 50,000 rapid diagnostic test (RDT) kits using funding from the UN Flash Appeal which was launched in June 2006. This accelerated the availability of both ACT and RDT in the country.

*Before the crisis, we have agreed to change the protocol to ACT. We needed some time to find the budget to buy ACT, so we have to wait. We are lucky because WHO donated 39,000 doses of ACT, we just received it last two weeks*.

MoH program implementer

### Vector Control and Insecticide Treated Nets (ITNs)

Vector control activities were planned by the Vector Control Working Group, comprising MoH and other development partners (NGOs and UN agencies). The Working Group also coordinated ITN distribution, fogging and larvaciding, and the training of health volunteers.

**Figure 3:**
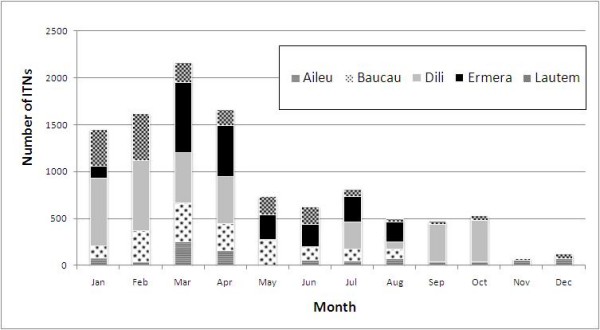
**Routine ITN distribution by the MoH in five selected districts, Timor-Leste, 2006**.

Prior to the crisis, routine ITN distribution strictly targeted pregnant women through antenatal care services, and children under 5 years of age. During the crisis, routine ITN distributions were briefly interrupted in some districts, notably in Dili in May and June 2006 (Figure 3). The MoH and NGOs diverted ITN stocks from routine programs to respond to the needs of IDPs.

Approximately 27000 ITNs were allocated to IDPs in three districts: Dili, Baucau and Viqueque, with priority being given to pregnant women and children under 5. About 90% of these ITNs were distributed to the IDPs in Dili. Nonetheless, some reservations concerning the effectiveness of ITN distribution and utilisation were expressed, although a detailed assessment was never undertaken:

*Bed net distribution in camps.. maybe it is not so much productive because it is very difficult to hang bed nets in the tents in a proper way.. this unfortunately gives a false security to the people*.

UN Agency

...*do people really use the bed nets that we distributed? This is what I see as a big dilemma, even we have given them education before giving bed nets, but we do not know whether these people really sleep under nets at night time, who would go to see them?*...

MoH Policy Maker

Disparities occurred in some districts and even in Dili some IDPs did not have access to ITNs. An IDP in a camp just outside Dili voiced his concerns regarding targeted and incomplete distribution:

*They distributed bed nets, but just for pregnant women and children.... only 14 families got bed nets, those ones came here first they received bed nets, those ones that came later, they have not received bed nets until now*.

IDP

Fogging and larvaciding were also applied during the emergency response, using health volunteers from IDP camps who were recruited and trained. The volunteers, however, were only active in the first month after training, apparently because many of them moved to other camps and hence the program was not sustained. At the time of interview in September 2006, fogging had been undertaken only once, in 33 IDP camps in and around Dili.

There was also disagreement over the use of *Malathion *to fog the camps. Some NGOs did not agree with its use for fogging because of the persistence of this chemical in the environment. These NGOs proposed indoor residual spraying as an alternative.

*Looking at insecticide spraying, I have to admit, MoH, HNI and CRS have different ideas of what should happen. In the end MoH did space spraying [fogging]. We were not excited about that, MoH did it, that was the decision of MoH. We were advocating residual spraying in the tents*.

International NGO senior officer

### Health promotion, inter-sectoral collaboration and training

Health promotion activities were undertaken in conjunction with ITN distribution and general medical assistance. Malaria was included in the key health promotion messages provided to the IDPs; key others focused on diarrhoea, immunisation, and hygiene and sanitation.

International peace keeping troops and the Australian Northern Territory Government provided assistance to the Vector Control Working Group, and also involved in the rainy season preparedness alongside other development partners. High risk camps for disease transmission especially diarrhoea, malaria and dengue had also been identified.

Some training activities could not be implemented because health staff were unable to travel to and from districts, resulting, for example, in cancelation of service training on microscopy.

*Because of the security, our colleagues from East they don't want to go to West to do malaria program and also for our colleagues from West don't want to go to the East*.

MoHProgram implementer

### The Global Fund and the malaria control program

The implementation of the Timor-Leste Global Fund for Malaria Program, funded through the Global Fund to Fight HIV/AIDS, Tuberculosis and Malaria, was delayed, however an agreement was reached to extend the implementation period until December 2006, at no extra cost. The reason for this delay was partly due to the crisis. Future funding opportunities were missed, however, because a new proposal was unable to be developed during this period of instability.

*the routine activities also get disturbed, for example, the Global Fund program, actually we have to finish it but because of this crisis we have to request for an extension until December. And we were not able to develop proposal for the Global Fund, next round*.

MoH Program Implementer

## Discussion

The malaria response during the crisis in Timor-Leste in 2006 was delivered by the MoH with the full support and collaboration of a range of development partners [17]. The intervention was rapidly organized, and the surveillance system in IDP camps in Dili promptly and effectively established. Despite the crisis disrupting routine ITN distribution and training programs, there were no major outbreaks of malaria detected during the period of instability.

Key questions covered in this discussion are: what factors helped to avoid a malaria outbreak during the crisis?; who was targeted in the interventions?; and to what extent were opportunities seized from the crisis response for improvement in malaria control in the long term?

### What factors helped to avoid a malaria outbreak?

The national malaria morbidity trends of 2006 showed no increase in malaria cases reported by the health system throughout the crisis. Malaria rates were even lower in Dili compared with the rest of the country which may well have been due to the early and coordinated multifaceted interventions. However, a slightly increasing trend in malaria diagnosis in Dili towards the end of 2006 and 2007 (the first malaria season after the crisis) could be explained by improved recording of cases and disruption of some of the control measures and supervision during the crisis. Trends in malaria incidence in Timor-Leste during the crisis presents a contrast with malaria in other conflict-affected countries such as in the Democratic Republic of Congo [8] in which malaria cases increased by 3.5-fold compared with the situation before the war. Significant increases in the national burden of malaria cases have also been reported from Afghanistan [9] and outbreaks have been reported in the highlands in Burundi [21].

In conflicts or in complex emergencies, factors that contribute to the increase of malaria morbidity and mortality include breakdown of health services and of malaria control programs, movement of people from low to high transmission areas, and environmental deterioration encouraging vector breeding [22,23]. The lack of any major malaria outbreak in Timor-Leste during the crisis may have been a result of the early malaria interventions through treatment and massive ITN distribution as well as the health promotion information provided to the IDPs in camps. Timing may also have been fortuitous as the crisis occurred toward the end of rainy season at which time malaria incidence trends typically decrease (see figure 1). Most people were displaced within Dili itself where access to nets, diagnosis, treatment and care continued to be present.

### Who was targeted in the intervention?

Since the crisis, much attention and resources have been devoted to the IDPs such as the intervention to distribute ITNs and the vector control activities for malaria and other vector borne diseases. The camp-focused intervention reflected the mobilization of ITNs from government (6000 nets) and NGOs (>21000 nets) to cover the needs of IDPs with about 90% of nets being provided to IDPs in Dili. The fogging and larvaciding also concentrated in IDP camps in Dili with volunteers recruited from the IDPs.

Prioritizing ITN distribution to pregnant women and children under five during the crisis was appropriate given that child mortality due to communicable diseases including malaria are often raised in conflict settings [24].

The multiple large IDP camps within the capital city (Dili) was somewhat unusual and presented a specific challenge requiring a comprehensive intervention plan. People in camps are at higher risk of mosquito bites because of improper shelter and overcrowding [25]. However, given that the camps in Dili were established not far from the surrounding communities, targeting only one side of the community (IDPs) and neglecting others (nearby communities) who share the same living environment (the city of Dili) is unhelpful. It was noted that the vector control interventions, particularly larvaciding and fogging, only targeted IDP camps, while community (non-IDPs) living within a few metres from IDP camps were not targeted with such interventions. Due to the proximity of the two communities, an outbreak of malaria or other vector borne disease would have impacted on both these sections of the Dili community. Therefore, in the future when displacement occurs in urban areas as seen in Dili in 2006, the malaria control interventions such as ITN distribution, vector control measures, and health promotion, should be targeted at the entire urban population rather than just those in IDP camps. Insecticide impregnated tents could also be usefully considered, especially given the difficulty of hanging nets in a tent structure.

### To what extent were opportunities seized from the crisis response to improve the malaria control program over the longer term?

Malaria cases in IDP camps were mostly diagnosed using a syndromic approach. There are two implications that arise; one is directly related to the IDPs as they did not gain access to better diagnosis; the other relates to the health system more generally which missed the opportunity to characterize the species of parasites causing malaria in Dili city. There was an opportunity available to undertake more reliable testing given that the IDPs were concentrated in camps. Although it may have been difficult to conduct microscopy examination in camps, access to other parts of the city including the hospital and available laboratory, were still present and logistical difficulties could have been overcome. The RDTs had been brought in to the country soon after the crisis but they were not used. Had the RDT tests been done, the parasite species could have been identified which would have been beneficial for both clinicians and health managers in forecasting appropriate antimalarial drug treatment needs.

The decision taken by the MoH and its partners during the crisis response considered health service delivery structure in IDP camps as a "temporary service" rather than as a "permanent structure". This may have prevented the provision of microscopy and RDT services in camp settings. As a result of this policy, a number of 24-hour fixed clinics had to be closed down in July 2006 with the intention that the IDPs can use health services available at Community Health Centres. The assumption was that having sophisticated health delivery at camp settings would only encourage people to stay in camps and thus could prolong the crisis. However, this highlights some of the limitations of seizing the momentum from the crisis to improve aspects of information and health system functioning.

The procurement of RDT and artemether-lumefantrine at that time of the crisis was justified because the risk assessment predicted potential disease outbreaks including malaria. Had the outbreak occurred at that time, the country was already prepared to respond.

In June 2007 the MoH replaced the previous protocol with a new protocol [26] which prescribed the use of RDT and ACT in malaria control in Timor-Leste. ACT has been shown to be effective in treating drug-resistant *falciparum *and *vivax *malaria in Papua, Indonesia [27]. It has been used in emergency situations across the globe, and is increasingly becoming standard treatment in malaria endemic countries [8]. The crisis generated some financial resources through the WHO component of the Flash Appeal, which was used to procure, in large quantities, both artemether-lumefantrine and RDT for Timor. Although the policy for changing the treatment protocol from sulphadoxine-pyrimethamine to ACT had been approved in June 2007 [26], the MoH had not iself procured ACT and RDT at that time but was able to use the ACT and RDT donated by WHO to facilitate the implementation of the newly approved treatment protocol. The crisis effectively facilitated the implementation of the policy in relation to malaria.

This crisis also provided an opportunity for Timorese health authorities to take charge of the operation, as demonstrated by the fact that health coordination structures, including the vector control and health promotion working groups were chaired by Timorese MoH staff as opposed to the earlier crisis in 1999, in which the NGOs had been the key players in delivering services and training [28]. In addition, no sidelining of local actors in responding to the crisis occurred, as happened in previous emergency responses in 1999 in Timor-Leste [29,30] or in Cambodia [31].

The crisis caused the loss of resources from the Global Fund as the country was unable to apply for a malaria grant in Round 6. Therefore, the government had to use its own resources to sustain the malaria control program. This highlighted a lesson for bilateral and multilateral donors to ensure flexibility in funding mechanisms in fragile states and unstable settings.

## Conclusions

The crisis response for malaria in 2006 brings both positive and negative lessons for future malaria control programs, particularly among urban displaced populations.

The positive side of the crisis response was that malaria control activities were collaboratively and rapidly organized by the MoH, UN Agencies and the NGO community, and was effectively coordinated by the MoH. The overall response conformed with the Roll Back Malaria Strategy and the crisis contributed to a positive longer term policy change. It was a Timorese-led intervention. The response is likely to have contributed to the lack of any major malaria outbreaks during the crisis.

The negative side of the crisis on malaria is that it disrupted training programs, impeded the MoH in attracting Global Fund resources, and the intervention was overly camp- focused rather than having an emphasis on the whole city.

Future crisis responses in which IDP camps are established in city areas, as was the case in Dili, deserve consideration. The intervention response must be planned beyond the IDPs alone, and adequate resources and expertise should be made available to assure a whole-of-city approach. Research should be advocated to improve malaria control in both normal and emergency circumstances in urban underserved areas in which displaced populations are present.

## Competing interests

We declare that we (the authors) have no competing interest in this article. Dr Nelson Martins (NM) is currently serving as the Minister for Health, Timor-Leste. At the time when this study was conducted, NM was a co-researcher involved in the study team.

## Authors' contributions

Joao Martins (JM) is a PhD scholar at the University of New South Wales. This study was part of his PhD thesis. JM was involved in conceptualizing this study, conducting data collection, data analysis, writing up the first draft of this paper and subsequently contributed to all stages of this paper until finalization.

Anthony Zwi (AZ) is supervisor for JM PhD studies. AZ led and coordinated the Timor-Leste Health Sector Performance and Resilience Study (Resilience Study), of which this study was a part. JM, NM and PK were also co-researchers in the Resilience study led by AZ. AZ contributed to conceptualizing this research and data analysis, and contributed to writing up and finalizing this paper.

Nelson Martins (NM) was as co-researcher for Resilience Study and contributed to data collection, study design and write-up.

Paul M Kelly (PK) is co-supervisor for JM's PhD studies. PK was involved in study design, data analysis and presentation, and all aspects of the write-up for publication.

All authors read and approved the final manuscript.
